# Deriving Multiple-Layer Information from a Motion-Sensing Mattress for Precision Care

**DOI:** 10.3390/s23031736

**Published:** 2023-02-03

**Authors:** Dorothy Bai, Mu-Chieh Ho, Bhekumuzi M. Mathunjwa, Yeh-Liang Hsu

**Affiliations:** 1 School of Gerontology and Long-Term Care, College of Nursing, Taipei Medical University, Taipei 110, Taiwan; 2 Gerontechnology Research Center, Yuan Ze University, Taoyuan 320, Taiwan

**Keywords:** motion-sensing mattress, patient fall, machine learning, care intervention, precision care

## Abstract

Bed is often the personal care unit in hospitals, nursing homes, and individuals’ homes. Rich care-related information can be derived from the sensing data from bed. Patient fall is a significant issue in hospitals, many of which are related to getting in and/or out of bed. To prevent bed falls, a motion-sensing mattress was developed for bed-exit detection. A machine learning algorithm deployed on the chip in the control box of the mattress identified the in-bed postures based on the on/off pressure pattern of 30 sensing areas to capture the users’ bed-exit intention. This study aimed to explore how sleep-related data derived from the on/off status of 30 sensing areas of this motion-sensing mattress can be used for multiple layers of precision care information, including wellbeing status on the dashboard and big data analysis for living pattern clustering. This study describes how multiple layers of personalized care-related information are further derived from the motion-sensing mattress, including real-time in-bed/off-bed status, daily records, sleep quality, prolonged pressure areas, and long-term living patterns. Twenty-four mattresses and the smart mattress care system (SMCS) were installed in a dementia nursing home in Taiwan for a field trial. Residents’ on-bed/off-bed data were collected for 12 weeks from August to October 2021. The SMCS was developed to display care-related information via an integrated dashboard as well as sending reminders to caregivers when detecting events such as bed exits and changes in patients’ sleep and living patterns. The ultimate goal is to support caregivers with precision care, reduce their care burden, and increase the quality of care. At the end of the field trial, we interviewed four caregivers for their subjective opinions about whether and how the SMCS helped their work. The caregivers’ main responses included that the SMCS helped caregivers notice the abnormal situation for people with dementia, communicate with family members of the residents, confirm medication adjustments, and whether the standard care procedure was appropriately conducted. Future studies are suggested to focus on integrated care strategy recommendations based on users’ personalized sleep-related data.

## 1. Introduction

Patient fall is a significant issue in hospitals [1,2,3]. Many falls are related to getting in and out of bed [4]. In Taiwan, patient falls were the second most common patient safety incident. In 2020, more than 60% of severe injuries caused by patient falls were related to getting in and out of bed [5]. Patients and medical organizations face increased lengths of stay and medical costs due to patient falls [6,7]. Hospitals have implemented many fall prevention measures, such as prevention procedures and identifying high-risk patients [7,8]. 

Technology solutions for bed-exit detection, such as movable sensing mats on top of the bed mattress, are increasingly used to prevent patient falls [9]. Umetani et al. [10] installed sensors on both sides of the comforter and wall in the bedroom to prevent bed falls. In addition to fall prevention, sensors are used to monitor in-bed activities for various purposes. Sensors have been placed beneath the legs of beds to determine how well residents sleep [11,12]. Sensors are also placed in mattresses for measuring ballistocardiograph signals during sleep [13,14] in long-term care. Sensors are integrated into pillows using wireless networks for sleep monitoring and gesture recognition [15]. Image processing is also used to monitor bed activities [16,17,18]; however, there have been concerns about the privacy of using image processing in sleep monitoring [19].

Machine learning algorithms are used to identify in-bed activities and categorize sleep. For example, hydraulic sensors were installed under mattresses to collect data [20,21]. Then, a convolutional neural network and a hybrid model of Long-Short Term Memory were applied [20] to classify three stages of sleep with an accuracy of 76%. Enayati et al. [21] classified combinations of four sleep postures with 93% accuracy using a simple neural network. Diao et al. [22] proposed a method for sleep posture recognition based on a dense, flexible sensor array and printed electrodes. They used Deep Residual Networks (ResNet) to distinguish sleep posture with an accuracy of 95%.

**Figure 1:**
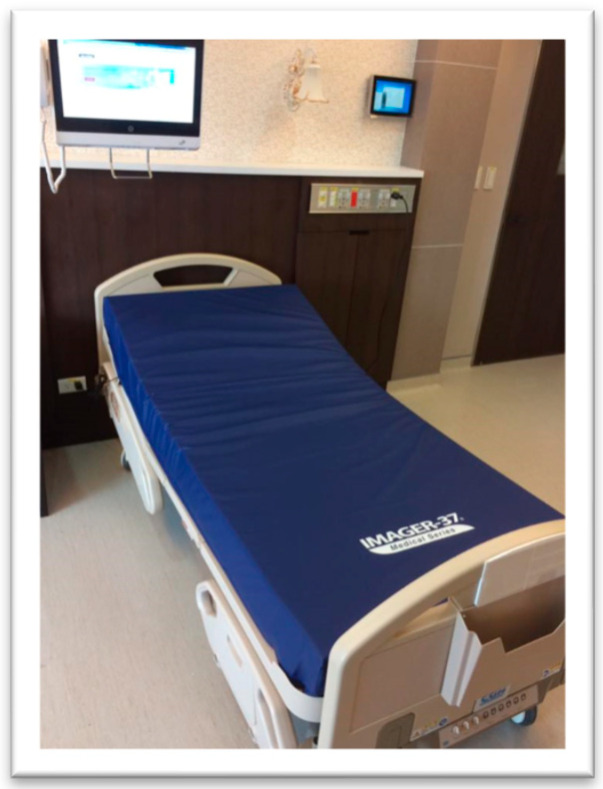
*Whiz*Pad is a foam mattress capable of motion sensing.

Bedridden patients are at high risk of developing pressure ulcers. Figure 1 shows a motion-sensing mattress (*Whiz*Pad^®^, SEDA G—Tech, New Taipei City, Taiwan) made of viscoelastic, temperature-sensitive polyurethane memory foam. In a study with 254 participants at risk of developing pressure injuries, recruited from the intensive care unit of a medical center, the use of the pressure-redistributing foam mattress was associated with an 88% reduced risk of pressure injury development [23].

**Figure 2:**
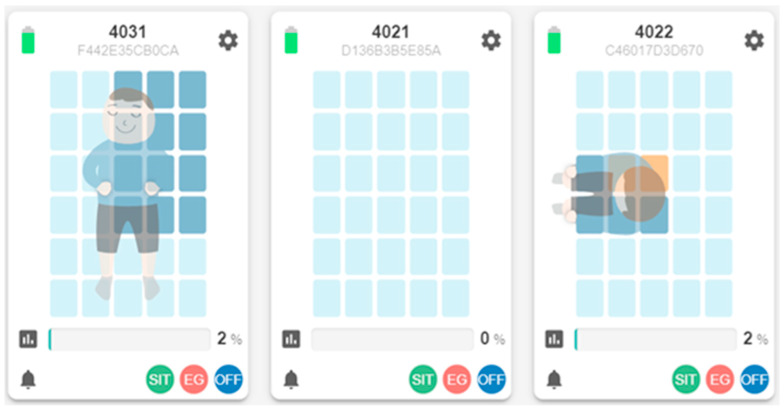
Real-time display of pressure pattern and in-bed posture.

As shown in Figure 2, the mattress contains 30 on/off pressure sensing areas. A machine learning algorithm is deployed on the mattress’s control box chip to identify the in-bed postures for capturing the patient’s intention to leave the bed. Training examples were collected from 31 participants of different weights and heights to identify three in-bed postures, including lying on the bed, sitting on the bed, sitting on the edge of the bed, and an empty bed. Each participant provided 30 examples, for a total of 930 examples, and 80 examples simulated an empty bed. Multilayer perceptron machine learning was used to create a posture identification model with an accuracy of 97.4% (10-fold cross-validation) and a ROC area of 99.7%. A smart mattress care system (SMCS) was built using IoT architecture to receive sensing and posture data from the mattress. The SMCS captures the patient’s intention to leave the bed from the sequence of the in-bed postures and sends out three bed-exit alerts [24]. When the patient at high risk of falling sits up on the bed, the SMCS sends the first early alert to the monitor at the nursing station or the mobile phone carried by the nurse, the second alert when the patient sits at the edge of the bed, and the third alert when the patient leaves the bed. Caregivers can adjust the alert function for patients with different fall risk levels. In a field trial in a medical center in Taiwan, with 25 nurses from a ward that had introduced the SMCS and 57 nurses from wards that used the traditional movable sensing mats, the average response time was significantly reduced (from 146s to 59s, *p* < 0.001). In the SMCS ward, no false bed-exit alarms were observed among 110 alarms. Comparatively, 42 (35%) of 120 alarms in traditional care practice were false bed-exit alarms (*p* < 0.001) and approximately 56% of nurses viewed the bed-exit alert as ignorable [25].

In addition to real-time status, caregivers in the nursing home or home also need long-term care related information about the residents, such as sleep quality, prolonged pressure areas, and living pattern analysis, and finally, support caregivers in precision care, help reduce their care burden, and enhance the quality of care. 

This study aimed to explore how sleep-related data derived from the on/off status of 30 sensing areas of a motion-sensing mattress can be used for multiple layers of precision care information, including wellbeing, status on the dashboard, and big data analysis for living pattern clustering. 

## 2. Methods

In this study, we describe how the SMCS is implemented to support caregivers and reduce care burden, while increasing the quality of care. The study also describes how multiple layers of personalized care-related information are derived from the motion-sensing mattress for long-term living patterns. In this section, we would first describe some basic functions of the sleep-related data in the system, which are the fundamental elements of further analysis for wellbeing, status on the dashboard, and big data analysis for living pattern clustering presented in the results. 

### Sleep Quality and Long-Term Living Pattern Analysis

**Figure 3:**
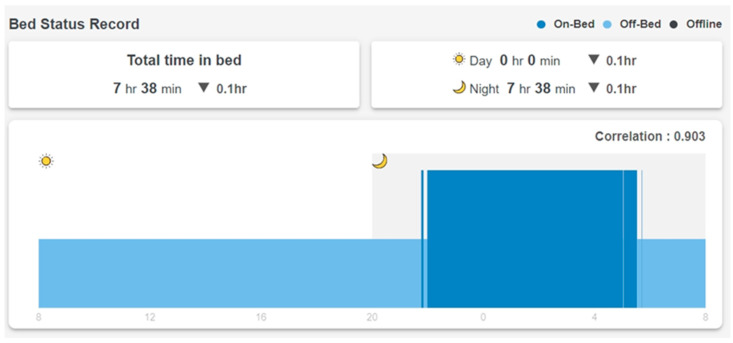
In-bed/off-bed record in 24 h.

In addition to the real-time display shown in Figure 2, the SMCS collects and displays long-term in-bed records. Figure 3 shows a resident’s in-bed/off-bed status in 24 h. Note that the horizontal time axis starts at 8 am and ends at 8 am the next day so that the overnight sleep of the resident can be displayed on the same chart. The vertical axis shows in-bed (higher bar) and off-bed (lower bar) status. Total time in bed during the 24 h is also displayed on top of the figure.

**Figure 4:**
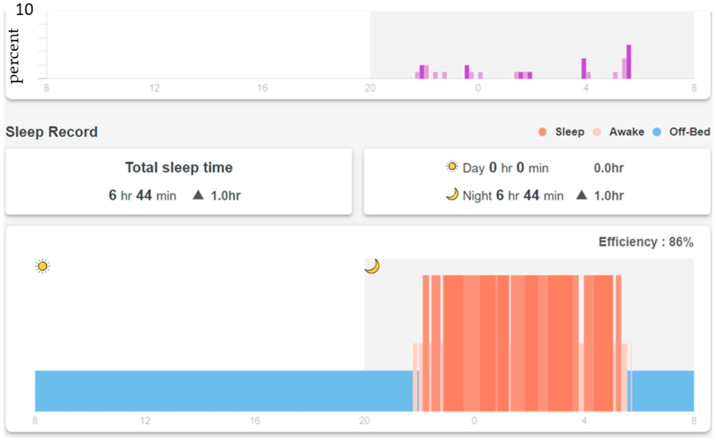
Movement counts and sleep record in 24 h.

When a resident lies on the bed, the motion-sensing mattress detects movement by comparing the on/off pressure pattern every second. The movement count is accumulated and sent to the SMCS every 30 s. Figure 4 shows movement counts and sleep status when the resident lies on the bed. The vertical axis is the percentage of time with movements per 10 min (total movement count divided by 600 s). A machine learning algorithm was trained using time series movement counts (240 s, 30 s per episode) as features and the sleep/awake scoring from polysomnography (PSG) as the label. Training examples were collected from five participants. Each participant provided overnight data (about 8 h) for a total of around 4000 examples. Multilayer perceptron machine learning was used to create a model with an accuracy of 89.0% (10-fold cross-validation). In particular, the sensitivity of awakeness is 69.2% [25]. As shown in Figure 4, the machine learning algorithm on the chip of the control box of the mattress classifies the in-bed time into sleep (high bar in Figure 4) and awake (medium bar in Figure 4). Total sleep time and sleep efficiency (86% in Figure 4) are also calculated and displayed on top of the chart.

**Figure 5:**
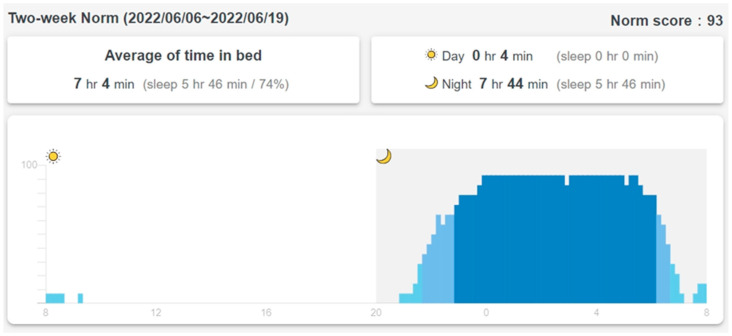
Two-week norm and norm score.

The SMCS calculates the average of 14 days of in-bed/off-bed status data and updates a two-week norm every Sunday (Figure 5). The vertical axis is the percentage of days that the resident is in bed. A 10-min time slot is considered “regularly in-bed” if the resident is in bed more than 70% (10 days out of the past 14 days) of the time (dark blue bars from 22:00 to about 7:00 in Figure 5). A 10-min slot is considered “regularly off-bed” if the resident is off the bed more than 70% of the time. The SMCS calculates a “norm score,” which is the percentage of “usually in-bed” plus “usually off-bed” time in the whole 24 h. The norm score for the past two weeks, as shown in Figure 5, is 93, which means that the resident has a very regular schedule every day in and out of bed. In Figure 3, the correlation factor of the daily in-bed/off-bed record with the two-week norm in Figure 5 is 0.903. Total time in bed is 0.1 h less than the two-week norm. The difference in total sleep time with the two-week average is also displayed in the sleep record in Figure 4.

The daily in-bed/off-bed status (Figure 3), daily sleep record (Figure 4), and two-week norm (Figure 5) were from the same participant for the purpose of this presentation, and they represent layers 3, 4, and 5 of personalized care information derived from the sensing data of the mattress. Intervention reminders are sent to the caregivers if the daily record deviates significantly from the norm.

At the end of the field trial, we interviewed the caregivers for their subjective opinions about whether and how the SMCS helped their work. The interview was held via an online meeting room for two hours due to the policy of the COVID-19 pandemic at that time in Taiwan. We invited four caregivers who had the experience of using SMCS for longer than six months to share their thoughts about SMCS. 

A descriptive analysis was used to examine participants’ sleep status such as in-bed hours during day and/or night, sleep hours during day and/or night, and sleep efficiency using mean, frequency, and percentage, depending on variables. Sleep norm related data, such as the in-bed norm hours and sleep norm hours, as well as norm score, was calculated using percentage related descriptive analysis with different ranges used according to different variables’ characteristics as described above. As for the clustering for different types of living patterns of residents in the nursing home, we used *k*-means clustering, which is an unsupervised learning algorithm in machine learning [26].

Study procedures were reviewed and approved by the Medical Ethics and Institutional Review Board of Taoyuan General Hospital, Ministry of Health and Welfare. Written informed consent was obtained from each participant or legal representative.

## 3. Results

### 3.1. Wellbeing Status on the Dashboard

**Figure 6:**
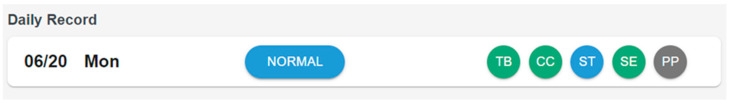
Dashboard for overall status.

This section describes the development of the Wellbeing Dashboard for *Whiz*Pad (WDW), in which the indices are combined, analyzed, and coherently presented. The indices are transformed into recommendations to support caregivers in planning personalized care programs. WDW uses the indices to provide caregivers with a weekly wellbeing status assessment of all older adults as well as individual users. As shown in Figure 6, there are five indices used in the dashboard:

- Single-day total time spent in bed (TB in Figure 6) compared to the two-week norm;
- Single-day correlation coefficient (CC) of the on/off bed pattern compared with the two-week norm;
- Single-day total sleep time (ST) compared to the two-week norm;
- Sleep efficiency (SE);
- Prolonged pressure (PP) time.

The first step in constructing the WDW is establishing a baseline for each resident. As described earlier, *Whiz*Pad provides the two-week norm, including average time in bed, on/off bed pattern, and average sleep time (Figure 5). The daily data is compared with the two-week norm to check if daily behavior deviates from the long-term pattern. Figure 3 shows that the total time in bed is 7 h and 38 min. The average time in bed is 7 h and 4 min (Figure 5). The difference is less than 1 h and a decision can be made by comparing the one-day data to the two-week norm. In the WDW, four levels are assigned to compare the difference between a single day’s total time spent in bed data and the two-week norm: Level 1 (±1 h), Level 2 (±1~2 h), Level 3 (±2~3 h), Level 4 (± over 3 h). According to the results in Figure 3, the difference in total time in bed is 34 min; thus, the user’s difference in total time in bed is at Level 1.

The correlation coefficient is also used in the dashboard to investigate whether changes in a single day sleep pattern are associated with the two-week norm. This correlation coefficient indicates whether the daily pattern conforms to the long-term pattern. Values between 0 and 0.3 indicate a weak correlation, values between 0.3 and 0.7 indicate a moderate correlation, and values between 0.7 and 1.0 indicate a strong correlation. In the WDW, four levels are assigned to the correlation coefficient: Level 1 (0.7 and above), Level 2 (below 0.7 and above 0.5), Level 3 (below 0.5 and above 0.3), and Level 4 (lower than 0.3). Figure 3 shows the correlation coefficient (0.903) of the on/off bed pattern with the two-week norm (Figure 5). The user’s correlation coefficient is at Level 1.

The Pittsburgh Sleep Quality Index (PSQI) is an effective instrument used to measure the quality and patterns of sleep in adults. Sleep efficiency is one of the seven components in evaluating sleep quality. The PSQI assigns a score of 0 to sleep efficiency, higher than 85%, 1 for 75%~84%, 2 for 65%~74%, and 3 for lower than 65% [27]. The WDW uses the same rules to assign the four levels: Level 1 (85% and above), Level 2, (between 84% and 75%), Level 3 (between 74% and 65%), and Level 4 (lower than 65%). The sleep efficiency for a user is shown in Figure 4 at 86%, indicating that the user’s sleep efficiency is at Level 1.

In the WDW, four levels are assigned to the difference in total sleep time from the two-week norm: Level 1 (± 1 h), Level 2 (±1~2 h), Level 3 (±2~3 h), and Level 4 (± over 3 h). Figure 4 also shows the total sleep time for the user (6 h 44 min), which indicates that the user’s difference in total sleep time is at Level 2.

**Figure 7:**
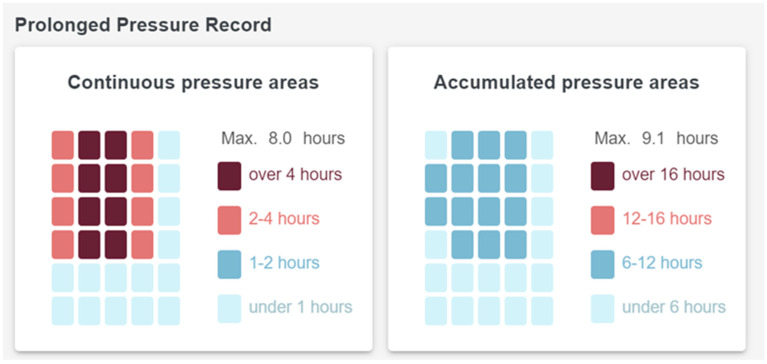
Prolonged pressure record.

Figure 7 shows the Prolonged Pressure Record provided by *Whiz*Pad, including the continuous pressure area and maximum time and the accumulated pressure area and maximum time. In common care practice, aged care facility residents at risk of pressure ulcers are repositioned at two-hour intervals. Therefore, in the WDW, four levels are assigned to continuous pressure time: Level 1 (below 1 h), Level 2 (above 1 h and below 2 h), Level 3 (above 2 h), and Level 4 (above 4 h). These indices only take effect if the total in-bed time exceeds 12 h.

Using Figure 6 as an example,

1. The difference in total in-bed time: Level 1 (<1 h);
2. Correlation coefficient: Level 1 (>0.7);
3. The difference in total sleep time: Level 2 (between 1 and 2 h);
4. Sleep efficiency: Level 1 (>85%);
5. Prolonged pressure time: not used (total in-bed time < 12 h).

**Figure 8:**
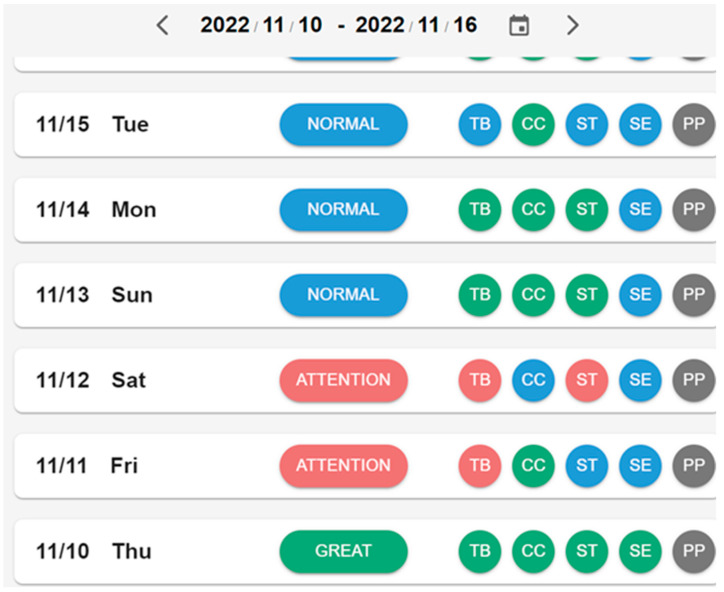
Wellbeing dashboard for seven days.

Figure 8 shows the overall status of a user during the course of one week. A user’s status is shown according to five levels of indices, including in-bed time (TB), correlation coefficient (CC), total sleep time (ST), sleep efficiency (SE), and prolonged pressure (PP). The indices’ final results are color-coded; green for “Great,” blue for “Normal,” red for “Attention,” and purple for “Abnormal.” The user is rated as “Normal” in Figure 6 because all related indices are at Level 1 or 2. The rules for the overall rating from the indices are as follows:

- Great: If all indices are in Level 1;
- Normal: If all indices are in Level 1 or Level 2;
- Attention: If one of the indices is at Level 3;
- Abnormal: If one of the indices is at Level 4;
- Off-Bed: If all indices have no values.

### 3.2. Big Data Analysis for Living Pattern Clustering

**Figure 9:**
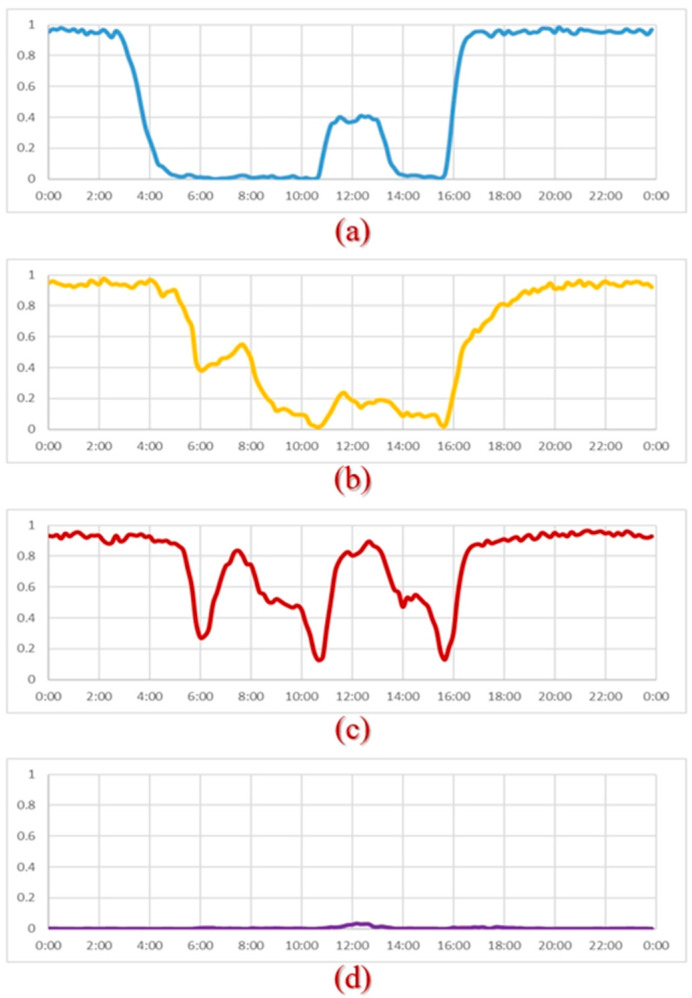
Daily in-bed/off-bed patterns of four types: (**a**) Regular (**b**) Free (**c**) Bedridden (**d**) Leave-home.

Twenty-four mattresses and the SMCS were installed in a dementia nursing home for a field trial. Residents’ on-bed/off-bed data were collected for 12 weeks from August to October 2021. The data were clustered into four types using *k*-means [27]. After confirming the living patterns of the residents with the caregivers, the four types of living patterns were named “Regular type,” “Free type,” “Bedridden type,” and “Leave-home type.” Figure 9 shows the averages of daily in-bed/off-bed records of all examples of the same type and the average in-bed time per day.

Residents of the free type have better activities of daily living (ADL) abilities and can freely get on and off the bed. Residents of the regular type are all wheelchair users. They are put on and off the bed by caregivers according to a regular schedule. Residents of the bedridden type lie in bed for an average of more than 18 h per day. As Figure 9 shows, they are helped off the bed briefly during breakfast (6:00), lunch (11:00), and dinner (16:00). If the resident leaves his or her bed, he or she usually heads to the hospital that day. 

**Figure 10:**
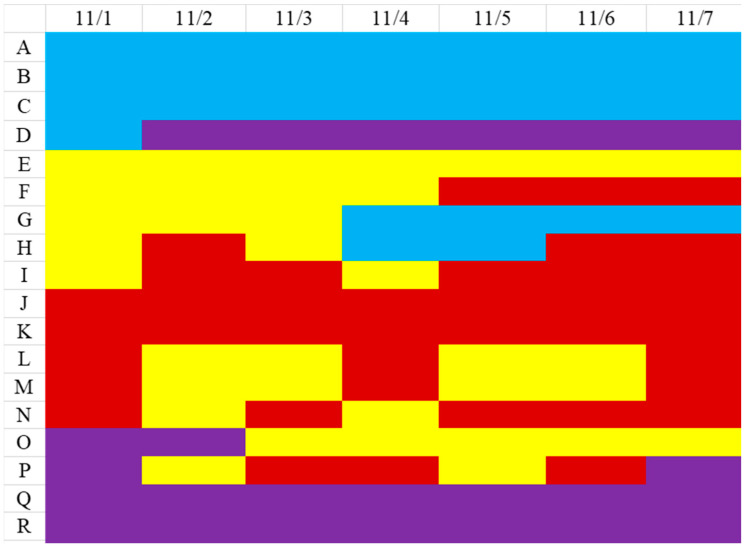
Living patterns changes of residents over 7 days.

The clustering model was used to classify November’s daily in-bed/off-bed status (Figure 10). The colors represent the four types of residents: blue for “regular”, purple for “leave-home”, yellow for “free”, and red for “bedridden”. For example, residents A, B, and C remained regular type for the whole week. Residents E, F, and G were free type. However, resident F changed to bedridden type after November 5th, and resident G changed to regular type after November 4th. The change in living patterns from one type to another often means a change in physical conditions.

The four living patterns provide a generic description of a resident’s condition in nursing homes and the 6th layer personalized care-related information derived from the sensing data of the mattress. The SMCS reminds caregivers of the need to intervene when a resident has a change in living pattern.

### 3.3. Evaluation by the Caregivers

**Figure 11:**
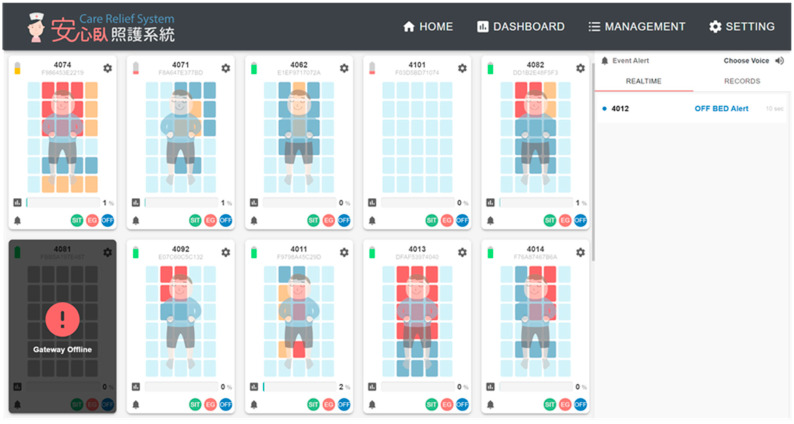
The screen displayed at the nursing station for the real-time status of the residents.

Figure 11 shows the screen displayed at the nursing station for the real-time status of the residents in the dementia nursing home in the field trial for 12 weeks. The three-stage bed-exit alerts (with audio) are shown on the right side of the screen. The color of the sensing area turns orange if the pressure lasts for more than one hour and turns red if the pressure lasts more than 2 h.

**Figure 12:**
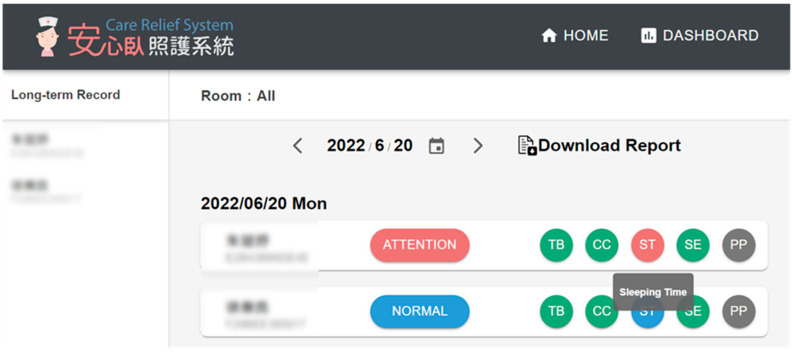
The list of records of all residents.

During the field trial, the caregivers were instructed to click “Dashboard” on the menu bar every morning to check the summary records of all residents in the past day (Figure 12), including the type of living pattern, total time in bed, and total sleep time, followed by the differences with the norm. The differences larger than two hours are displayed in red. The caregivers were asked to click on the personal record in red to view the detailed figures.

At the end of the field trial, we interviewed the caregivers for their subjective opinions about whether and how the SMCS helped with their work. The main items that these caregivers mentioned about SMCS were listed as below:

- The intervention reminders spoke for the residents with dementia who were not able to express their discomforts clearly;
- The figures and data from SMCS helped with communication with the family members of the residents;
- The sleep record helped to confirm the medication adjustments for the residents;
- The long-term records helped confirm whether the standard care procedure is correctly done, such as body turns every two hours for bedridden residents.

## 4. Discussion 

The present study investigates how multiple layers of personalized care-related information can be derived from a motion-sensing mattress for long-term living patterns. The SMCS is designed to reduce care-related incidents that are associated with aging and release care burden for caregivers. The SMCS is equipped with care-specific alerts that are sent directly to assigned caregivers to increase the efficiency of care.

Falling among elderly patients is one of the common incidents addressed by the proposed system. Preventing patient falls and injuries associated with older adults requires knowledge of falls and related injuries. Studies have shown that falls among older adults are related to getting in and out of bed [4,28,29]. A study by [4] also reveals that falls are associated with patient confusion. Patients who are bedridden are at high risk. Hospitals and nursing homes face a challenge when it comes to integrating bed-exit alarm systems into their facilities. Traditionally, patient care systems use alarms as emergency calls whenever the patient needs assistance. As patient care alarms in traditional care facilities are not specific, caregivers cannot distinguish between the kind of assistance needed for the patient. They rely on their experience with patients to determine whether the alarm is of high priority. This can lead to many alarms being ignored by caregivers [25] and can also add to caregivers’ burden when multiple patients are cared for by the same caregiver. Incorporating bed-exit alarm systems with extended communication features into mattresses and other parts of nursing beds can create a substantial market in the healthcare industry. The SMCS can also alert caring relatives when a family member’s status changes. Caring family members will be able to feel confident about their relatives’ safety with a reliable care system. It is also possible to assist relatives in fulfilling their responsibility as caregivers and be able to socialize by engaging in relaxing activities. According to our study, it is possible to reduce falls through the use of SMCS.

Sleep quality is an essential part of the care of older adults. According to studies, sleep quality in terms of duration and consolidation changes with aging [30,31]. In older adults, reduced cognitive function and dementia can be attributed to poor sleep quality [32]. Research on how sleep quality affects cognitive decline in older adults has become critically important, given the world’s aging population [33]. It is difficult to evaluate sleep quality empirically due to its complex nature. As a result, the validity of current and future research efforts depends heavily on the methods used to quantify sleep quality parameters. Historically, sleep quality has been assessed using a variety of methods, including subjective measures such as the Consensus Sleep Diary and Pittsburgh Sleep Quality Index, as well as objective measures like polysomnography and actigraphy.

PSQI is commonly used as a measure of sleep quality in studies. Furthermore, the PSQI may not be relied on to measure sleeping habits for older adults because it requires their cognitive capacity to reflect on the previous month. Further study is needed to determine the PSQI’s validity among older adults. Using in-bed sensor data, the current study presents an alternative measure for assessing sleep quality in older adults. By comparing the different measures of assessment with the previous 2-week data for the same person, we are able to identify changes in their sleeping habits. Time-on-bed and sleep records are measured by the SMCS as part of the sleep monitoring assessment for older adults. The SMCS collects, displays, and compares 24 h of sleep data with long-term in-bed records. Caretakers can use this data to draw conclusions about the elderly’s sleep and health conditions. In cases where deviations are reported by the system in relation to the data collected from older adults, further action can be taken on time, thus enhancing user wellbeing.

The bed is often the central care unit in hospitals, nursing homes, and homes. The information needs of nurses and formal and informal caregivers are pretty different. Rich personalized care information can be derived from sensing data from the bed. This study describes the multiple layers of personalized care-related information derived from the on/off status of 30 sensing areas of the motion mattress, summarized below:

1. Layer 1: On/off status of 30 sensing areas;
2. Layer 2: In-bed posture;
3. Layer 3: Daily in-bed/off-bed records;
4. Layer 4: Daily sleep and prolonged pressure records;
5. Layer 5: Two-week norm;
6. Layer 6: Four types of living patterns.

For every patient, a two-week norm is calculated to compare their daily in-bed pattern and identify deviations that may indicate the need for intervention. A dashboard is a graphic user interface that provides caregivers with a central entry point for descriptive analyses of the users’ overall wellbeing. Caregivers quickly identify clients who need specific attention and then check into the detailed data of the indices in order to make decisions about the care and support for the client. 

However, this study has some limitations. First, in this study, we only included people with dementia as the users of the SMCS for living pattern clustering. This would limit the generalization of the study findings to other groups of older adults in need of precision care. Second, we only investigated the fundamental usage of sleep-related data derived from the on/off status of 30 sensing areas of a motion-sensing mattress. For example, we tried to use these original sensors’ data for wellbeing status on the dashboard and living pattern clustering. This only addressed the basic concern of caregivers without any recommendations for nursing strategies. On the basis of users’ personalized sleep data, we should further investigate integrated care strategy recommendations. Lastly, we collected user experience data from four caregivers to discover how the SMCS helped them in their work. Since Taiwan was dealing with the COVID-19 pandemic at that time, the interviews took place via an online meeting room for two hours without a thorough interview and interaction. Moreover, we could not get enough nurses to give their subjective opinion. In addition, nurses and patients are both involved in patient care systems, but we focused primarily on nurses’ perspectives and experiences. In the future, data should also be collected from patients, as well as some other stakeholders, such as the managers of the nursing home and patients’ families. 

## 5. Conclusions

This study used sleep-related data derived from the on/off status of 30 sensing areas of a motion-sensing mattress for multiple layers of precision care information including wellbeing status on the dashboard and big data analysis for living pattern clustering, including real-time in-bed/off-bed status, daily records, sleep quality, prolonged pressure areas, and long-term living patterns. A 12-week field trial was conducted in a dementia nursing home with 24 mattresses set up for data collection as well as caregivers’ user experience. The caregivers’ main responses included that the SMCS helped caregivers notice the abnormal situation for people with dementia, communicate with family members of the residents, confirm medication adjustments, and whether the standard care procedure was appropriately conducted. The ultimate goal of SMCS is to support caregivers for precision care, reduce their care burden, and increase the quality of care. Future studies are suggested to focus on integrated care strategy recommendations based on users’ personalized sleep-related data.

## Data Availability

Data is unavailable due to privacy issues.
